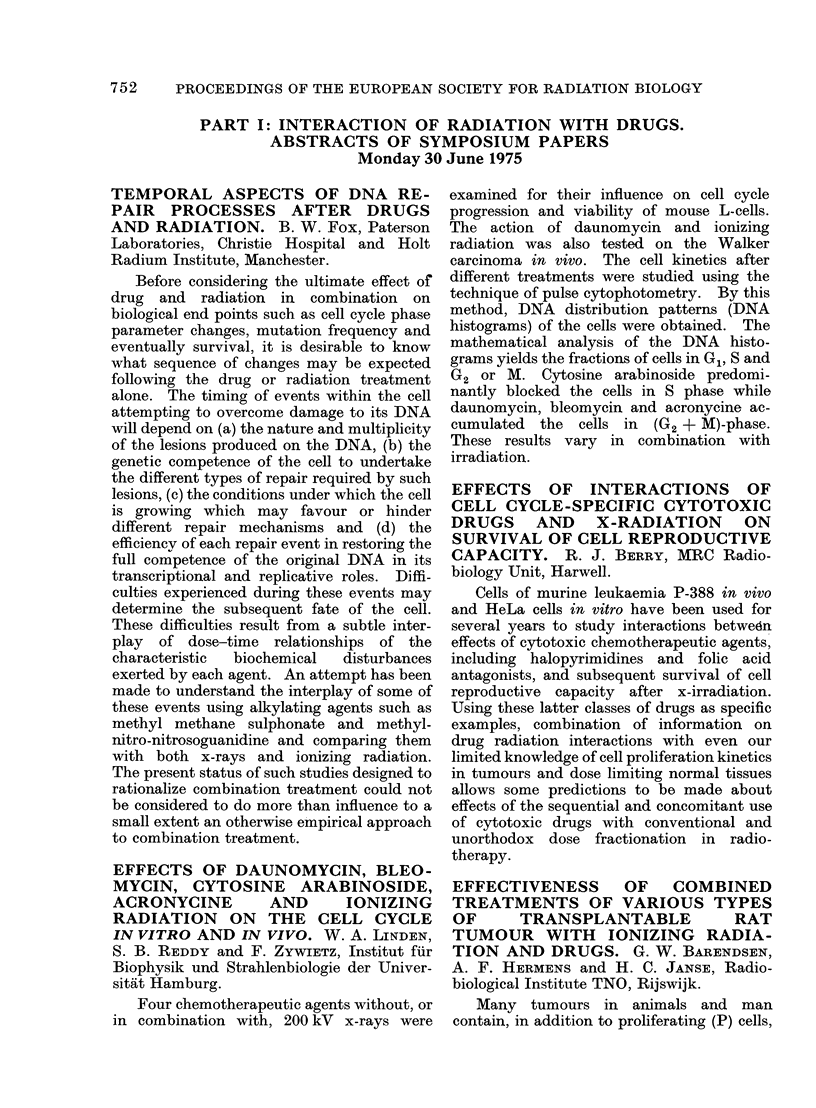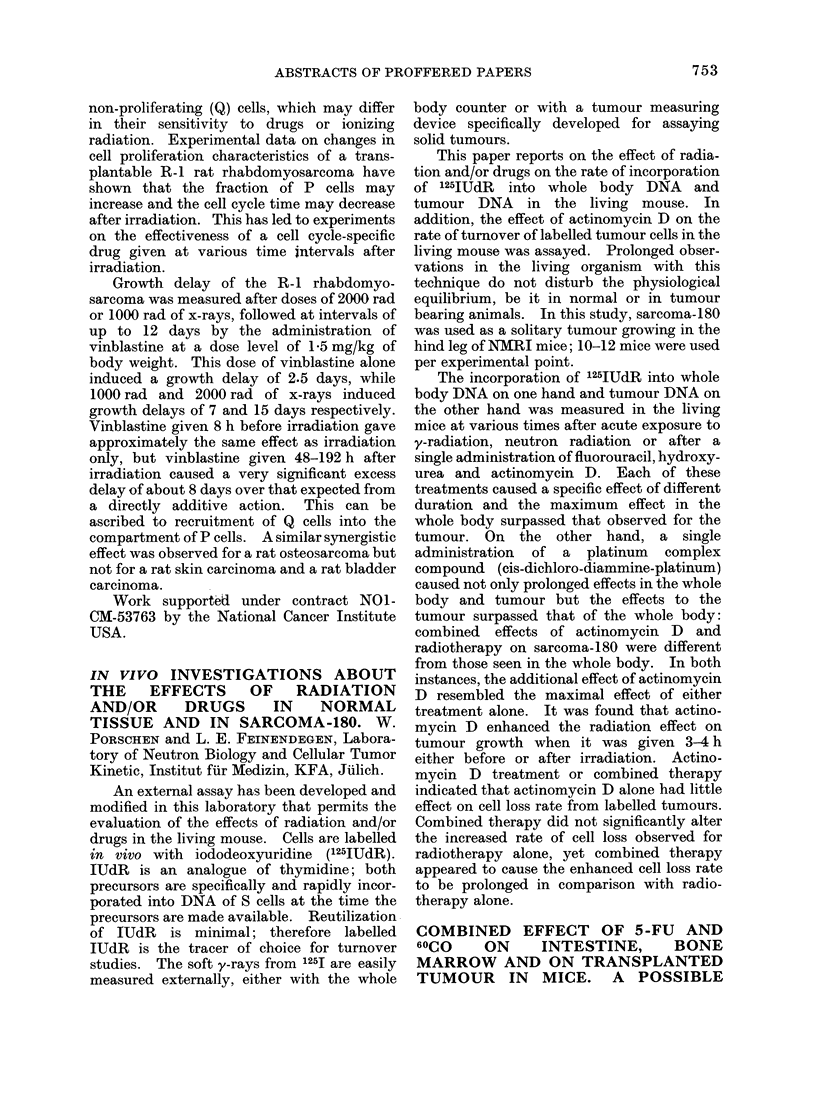# Proceedings: Effectiveness of combined treatments of various types of transplantable rat tumour with ionizing radiation and drugs.

**DOI:** 10.1038/bjc.1975.295

**Published:** 1975-12

**Authors:** G. W. Barendsen, A. F. Hermens, H. C. Janse


**EFFECTIVENESS OF COMBINED TREATMENTS OF VARIOUS TYPES OF TRANSPLANTABLE RAT TUMOUR WITH IONIZING RADIATION AND DRUGS.** G. W. Barendsen, A. F. Hermens and H. C. Janse, Radiobiological Institute TNO, Rijswijk.

Many tumours in animals and man contain, in addition to proliferating (P) cells, non-proliferating (Q) cells, which may differ in their sensitivity to drugs or ionizing radiation. Experimental data on changes in cell proliferation characteristics of a transplantable R-1 rat rhabdomyosarcoma have shown that the fraction of P cells may increase and the cell cycle time may decrease after irradiation. This has led to experiments on the effectiveness of a cell cycle-specific drug given at various time intervals after irradiation.

Growth delay of the R-1 rhabdomyosarcoma was measured after doses of 2000 rad or 1000 rad of x-rays, followed at intervals of up to 12 days by the administration of vinblastine at a dose level of 1·5 mg/kg of body weight. This dose of vinblastine alone induced a growth delay of 2.5 days, while 1000 rad and 2000 rad of x-rays induced growth delays of 7 and 15 days respectively. Vinblastine given 8 h before irradiation gave approximately the same effect as irradiation only, but vinblastine given 48–192 h after irradiation caused a very significant excess delay of about 8 days over that expected from a directly additive action. This can be ascribed to recruitment of Q cells into the compartment of P cells. A similar synergistic effect was observed for a rat osteosarcoma but not for a rat skin carcinoma and a rat bladder carcinoma.

Work supported under contract NO1-CM-53763 by the National Cancer Institute USA.